# Quantifying photothrombotic ischemic volume in the intact brain using light sheet microscopy

**DOI:** 10.1117/1.NPh.13.1.015009

**Published:** 2026-02-03

**Authors:** Jessika Royea, Catherine Albert, Valérie Daigneault, Patrick Chary, Ismaël Bachand, Jean-François Bouchard, Gergely Silasi, Matthieu P. Vanni

**Affiliations:** a Université de Montréal, École d’Optométrie, Montréal, Québec, Canada; b Université de Montréal, Centre Interdisciplinaire de Recherche sur le Cerveau et l’Apprentissage, Montréal, Québec, Canada; c University of Ottawa, Department of Cellular and Molecular Medicine, Ottawa, Ontario, Canada

**Keywords:** light sheet fluorescent microscopy, ischemic volume, stroke, photothrombosis

## Abstract

Traditional methods for assessing photothrombotic stroke rely on *in vivo* imaging techniques or *ex vivo* histological analyses. Unlike *in vivo* modalities such as magnetic resonance imaging (MRI), light sheet fluorescence microscopy (LSFM) provides cellular-level high-resolution imaging without motion artifacts and can capture fine-scale morphological features of infarcts. In addition, compared with conventional histology, LSFM preserves organ integrity as the entire brain is imaged without the need for serial sectioning, thereby enabling accurate volumetric reconstruction of ischemic lesions. Here, we introduce an in-depth semi-automated method for reliable quantification of stroke volume using LSFM in optically cleared whole mouse brains following photothrombotic stroke. We demonstrate that the infarct can be delineated via endogenous autofluorescence, providing a reproducible and robust method for ischemic volume assessment. Our data show that LSFM-based stroke volume measurements are strongly correlated with *in vivo* laser speckle contrast imaging, *in vivo* MRI, and cresyl violet histology measures of stroke volume. Moreover, we show that the ischemic core remains autofluorescent regardless of the tissue preparation method, supporting the applicability of LSFM for both freshly processed and long-term stored samples. Overall, our findings validate LSFM as a reliable, versatile, and powerful alternative method for stroke volume quantification, offering significant advantages for experimental stroke research.

## 1 Introduction

Stroke is a major cause of mortality worldwide and remains a primary cause of disability, resulting in significant personal, social, and economic burden. Despite a large number of drugs and therapies tested over the last decades, there is currently no effective strategy to completely prevent or cure the disease. Thrombolytic therapy and thrombectomy^1^ are the only currently approved clinical interventions, but both need to be performed within a limited time window after symptom onset.^2^ For this reason, there is an urgent need to develop new treatments for acute ischemic stroke. Preclinical models are therefore essential and require standardized, reliable experimental protocols to assess therapeutic intervention on stroke outcomes, including stroke volume.

Rodent models are the current standard for assessing ischemic stroke treatment strategies. Photothrombosis, a commonly used method, induces stroke in a controlled manner by photoactivating an intravascular photosensitive dye to cause targeted vascular occlusion.^3^ This stroke model is often used to study stroke pathology, therapeutic interventions, as well as brain plasticity and recovery mechanisms. A crucial component in assessing stroke outcomes is the ability to accurately quantify and localize the injury, thus enabling the assessment of treatment efficacy and aiding our understanding of the progression and impact of stroke.

In rodents, the ischemic lesion resulting from photothrombosis can be visualized and quantified *in vivo* using imaging techniques such as magnetic resonance imaging (MRI)^4^ or via laser speckle contrast imaging (LSCI).^5^ Although informative, quantifying photothrombotic stroke volume using MRI presents limitations due to the complexities of live imaging, including resolution limits of imaging techniques, tissue contrast, movement artifacts, and access to this advanced imaging equipment.^6^^,^^7^ Meanwhile, LSCI, a wide-field non-invasive imaging technique based on the analysis of infrared light signals scattered off of moving red blood cells, is a useful tool to confirm the presence of stroke.^5^ A considerable limitation of LSCI is the shallow tissue penetration depth and overall resolution; therefore, it is ideally paired with *ex vivo* histological analyses that complement these limitations. Although these techniques provide valuable information, at early time points, MRI primarily reflects a measure of the ischemic infarct possibly exaggerated by significant vasogenic and cytotoxic edema,^8^ whereas speckle imaging provides an indirect measure of changes in cerebral blood flow,^9^ both of which may not accurately represent the true lesion size.

Traditionally, the gold standard approaches for quantifying lesion volume in stroke studies are through tissue staining with triphenyltetrazolium chloride or cresyl violet to highlight the areas of infarction.^10^^–^^13^ Stained tissue is subjected to microscopic examination and coupled with image analysis software to quantify the area of damage across sequential sections while considering the thickness of each slice. However, histological processing is labor-intensive and can be subject to variabilities in staining intensity and interpretation, potentially leading to errors in lesion size quantification. Lesion volume is often calculated as the sum of the infarct areas across sections, multiplied by the distance among sections, thus providing an estimate of lesion volume. Although histology remains the gold standard for lesion size quantification, it requires registration of regions of interest to brain atlases, whereby such registration is more feasible for intact tissue volumes, in which the relative spatial relationship among anatomical structures is preserved. Indeed, these techniques are more difficult to integrate into automated lesion localization pipelines, limiting their utility in studies requiring precise anatomical mapping.

Light sheet fluorescence microscopy (LSFM) of optically cleared brain tissue enables high-resolution, three-dimensional imaging of the intact rodent brain. Unlike traditional histological methods, LSFM preserves anatomical continuity and allows for accurate volumetric quantification. Following photothrombosis, infarcted brain tissue exhibits endogenous autofluorescence that correlates with cresyl violet staining,^10^ providing a label-free approach to delineate ischemic lesions. In this study, we present a rapid and reproducible semi-automated method for quantifying infarct volume using LSFM-based detection of ischemia-induced autofluorescence in cleared mouse brains. Our approach maintains the stroke lesion within the intact brain, captures its three-dimensional morphology, and avoids artifacts associated with sectioning. Moreover, we demonstrate that infarct volumes are consistent across imaging orientations and strongly correlate with conventional imaging modalities, validating LSFM as a robust and flexible platform for stroke volume quantification and multiparametric analysis in preclinical research.

## 2 Methods

All experiments conducted in this study were subjected to ethical approval from the University of Montreal and the University of Ottawa and meet the guidelines of the Canadian Council on Animal Care.

### 2.1 Surgical Implant of Cortical Window and Head Fixation Bar

Cranial windows were used in a subset of mice to enable laser speckle contrast imaging. Mice were anesthetized with isoflurane (3% in pure oxygen for induction, maintained at 1.5% to 2% after skin incision; Fresenius Kabi Canada Ltd., Toronto, Canada). Body temperature was maintained at 37°C±0.5°C using a heating pad and monitored by a rectal thermometer (Harvard Apparatus, Holliston, Massachusetts, United States). Mice received a subcutaneous injection of carprofen (0.5  mg/mL in saline, 0.01  mL/g; Rimadyl, Best Buy Medical Supplies Inc., Surrey, Canada), and their eyes were lubricated (Systane). The scalp was shaved and cleaned with alcohol followed by providone-iodine (TEVA Canada, Whitchurch-Stouffville, Canada), and a local anesthetic (lidocaine, 0.2 mL) was injected subcutaneously. The skull was exposed, and the surrounding skin was affixed with cyanoacrylate (Vetbond). Clear dental cement (C&B Metabond) was applied to the skull, and a glass coverslip was secured in place. A titanium head bar (Labeo Technologies Inc., Montreal, Canada) was affixed posterior to the cranial window to allow head fixation during imaging. Post-surgery, mice received 0.5 mL of subcutaneous saline and were placed in a warmed recovery cage until ambulatory (∼30  min). After 48 h of monitoring, they were returned to their home cage. Carprofen was administered daily for 2 days and extended to 3 days if pain was evident (0.01  mL/g).^11^

### 2.2 Laser Speckle Contrast Imaging

Speckle imaging, which visualizes interference pattern distortions from laser light scattered by blood flow,^5^^,^^12^ was used to assess cerebral blood flow before and 2 days after stroke induction. LSCI was performed on n=27 mice using the LightTrack OiS200 system (Labeo Technologies Inc.).^5^ The cortex was illuminated with a 785-nm infrared laser (7.2 mW), and images were captured with a sCMOS camera for 30 s (1024×1024 resolution over a 10×10  mm area) at 10 Hz with a 10-ms exposure.^5^ Speckle contrast (k = standard deviation/mean intensity) was calculated in MATLAB (MathWorks, Natick, Massachusetts, United States). Contrast maps were generated to confirm the location and size of the induced stroke.

### 2.3 Photothrombotic Stroke Induction

**Fig. 1:**
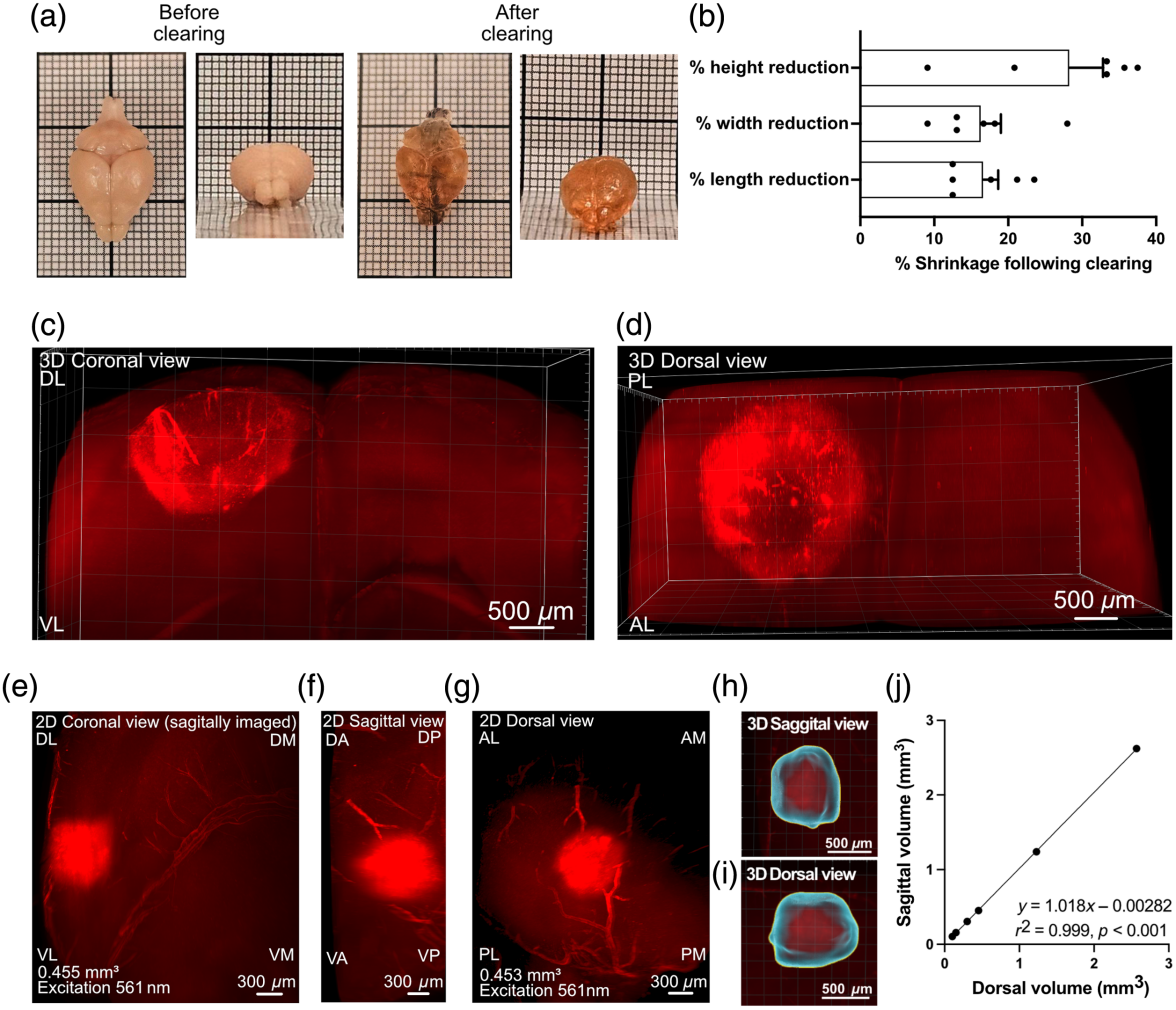
Brain clearing enables 3D quantification of stroke lesion volume using LSFM. (a) Representative images of mouse brains before and after tissue clearing, showing increased transparency post-clearing. Smallest squares are 1×1  mm, and bold squares are 10×10  mm. (b) Representative percent difference in reductions in brain height, width, and length following tissue clearing, as shown across the three measured dimensions. (c)–(i) Representative images of autofluorescent infarcts were acquired using a 561-nm fibered laser combined with a 600/50-nm band-pass emission filter. (c)–(d) 3D-rendered coronal and dorsal views of a cleared mouse brain showing the ischemic lesion based on intrinsic autoflorescence. 2D coronal (e), sagittal (f), and dorsal (g) projections of the same lesion, yielding nearly identical volume estimates (0.455 and $0.453\;{\mathrm{mm}}^{3}$, respectively). (h) and (i) 3D reconstructions of the infarct from sagittal and dorsal perspectives confirm spatial consistency and lesion boundary overlap. (j) Volumetric measurements of the infarct from sagittal and dorsal planes are highly concordant ($r^{2}=0.999$, p<0.001), validating reproducibility and robustness of lesion quantification across imaging orientations. Scale bars: (c) and (d) 500  μm, (e)–(g) 300  μm, and (h) and (i) 500  μm. Orientations: D, dorsal; V, ventral; A, anterior; P, posterior; L, lateral; M, medial (Video 1, MP4, 16.77 MB [URL: https://doi.org/10.1117/1.NPh.13.1.015009.s1]).

For cranial window mice (n=40), a 2-day habituation to head fixation (via titanium bar; Labeo Technologies Inc.) was performed. Strokes were induced in the right or left motor or visual cortex based on Allen Brain Atlas coordinates [Motor AP 0, ML 1.5; Visual AP 3.5, ML 2.4; Fig. 1(b)],^14^^,^^15^ avoiding large vessels and sutures. A 532-nm green laser [6 (small) or 11 mW (large), ∼1  mm beam diameter] was targeted via the galvo mirrors in an optical imaging system (Labeo Technologies Inc.). Mice were injected intraperitoneally (10  mL/kg) with Rose Bengal (0.015  g/mL; Sigma Aldrich, 198250-5G, St. Louis, Missouri, United States). After 2 min of circulation, the targeted area was illuminated for 8 or 13 min. The Rose Bengal–light interaction generated reactive oxygen species, triggering localized thrombosis. Following stroke induction, the mice were removed from fixation and monitored daily until euthanasia at 10 days post-stroke. For MRI mice, the procedure was similar, whereby mice were anesthetized with isoflurane (5% induction and 2% maintenance) administered meloxicam subcutaneously (5  mg/kg), the head was shaved, and the skull was exposed. A 10-mg/mL Rose Bengal solution [phosphate buffer saline (PBS)-based] was injected (100  mg/kg IP) and allowed to circulate for 2 min prior to turning on the laser. A 532-nm green laser ($7\;\mathrm{mW}/{\mathrm{mm}}^{2}$, 1.2 mm diameter) was positioned over the right hemisphere (1.5 mm lateral to the bregma) for 13 min. Mice recovered in heated cages and were monitored daily until euthanasia at 7 days post-stroke.

### 2.4 Magnetic Resonance Imaging

Infarct volume for n=4 mice was determined *in vivo* 7 days post-photothrombosis using a small animal magnetic resonance scanner (Agilent MR901 7-Tesla General Electric, Santa Clara, California, United States) at the University of Ottawa. Mice were anesthetized with isoflurane (5% induction, 2% maintenance, Fresenius Kabi Canada Ltd.), and their respiratory rate was monitored using a small animal monitoring and gating system (Small Animal Instruments, Inc., Stony Brook, New York, United States). Images were acquired with a T2-weighted fast spin echo pulse sequence, with the following parameters: slice thickness, 0.2 mm; field of view, 2 cm; matrix, 256×256; echo time, 39 ms; repetition time, 15,000 ms; and average imaging time of 32 min. The stroke lesions, characterized by marked hyperintensity, were outlined using ImageJ (National Institute of Health Research, Bethesda, Maryland, United States), and the volumes were determined by multiplying the infarcted area for all coronal sections by the magnetic resonance slice thickness.

### 2.5 Tissue Preparation

Tissue was prepared in three different ways: (i) paraformaldehyde (PFA) post-fixed and maintained at 4°C, (ii) PFA perfused and maintained at 4°C, and (iii) PFA perfused and frozen at −80°C, to validate the ability to assess stroke volume in archived tissue. The first subset of mice was anesthetized with isoflurane (3% in pure oxygen), and a subset of mice (n=9) was decapitated, their brains extracted, post-fixed in 4% PFA (EMS, 15710-S) for 48 h at 4°C, and stored at 4°C in 0.1 M PBS. A subset of these mice (n=4) received a cardiac injection of Evans blue (EB, 2%, 100  μL) prior to decapitation to indirectly label the cerebrovasculature. A second subset (n=4) was anesthetized with isoflurane (3% in pure oxygen) and perfused intracardially with 4% PFA, and the brains were post-fixed (4% PFA, overnight, 4°C) and stored at 4°C in 0.1 M PBS. The remaining mice (n=27) were anesthetized with isoflurane (2% in pure oxygen) and perfused intracardially with 4% PFA, and the brains were post-fixed (4% PFA, overnight, 4°C), cryoprotected (48 h, 30% sucrose, 4°C), flash frozen in isopentane −30°C, and stored at −80°C.

### 2.6 Tissue Dehydration, Delipidization, and Clearing

The brains were cleared using a modified version of the iDISCO protocol. Briefly, the brains stored at −80°C underwent three 30-min PBS washes prior to dehydration, whereas the brains stored at 4°C in PBS did not require additional washes. Mouse brains were dehydrated via incubations in an increasing series of methanol/water washes: 20%, 40%, and 60% (2 h at each stage, shaking, at room temperature) and transferred to 60% methanol for an overnight wash at room temperature. The following day, the brains were washed in methanol/water dilutions of 80% and 100% (2 h at each stage, shaking, at room temperature) and transferred to 100% methanol for an overnight wash at room temperature. The brains were then delipidized in 66% dichloromethane (Sigma, 270997)/33% methanol (Fisher, A412SK) for 48 h (4°C, shaking). Following delipidization, the brains were washed in 100% DCM three times for 15 min and incubated in dibenzyl ether solution (Sigma, 108014) until the samples became transparent and stored at 4°C. Importantly, to minimize air induced oxidation, which leads to a redish hue within brain tissue, Eppendorf tubes were filled completely with dibenzyl ether to avoid brain contact with air.

### 2.7 Light Sheet Data Acquisition and Image Processing

Cleared whole brains were imaged using an Alpha3 commercial light sheet microscope (Phaseview/Olympus). Briefly, the brains were glued (RTV Silicone 5145, Henkel-LOCTITE) to a transparent platform and submerged in a chamber filled with ethyl cinnamate (Alfa Aesar, A12906-36). Optical imaging of the cleared brains was performed using the 561-nm fibered laser paired with a 600/50-nm band-pass emission filter to collect the ischemic autofluorescence. EB vascular labeling was assessed using the 638-nm fibered laser paired with a 670/50 band-pass filter. Optical imaging was additionally performed using the 445-, 448-, 561-, 638-, and 730-nm fibered lasers paired with their respective band-pass emission filters to confirm autofluorescence throughout the spectra (Fig. S1 in the Supplementary Material). Images of the brain were acquired with an XLFluor 4× 0.28 NA objectives (Olympus) with 10- to 15-μm steps in the axial direction. To render the brain images, z-stacks and XY-tiling of our region of interest were performed using the QtSPIM software. Once QtSPIM XML files were generated, they were converted using the IMARIS file converter software [note: X, Y, and Z parameters can be read by opening the XML file in Fiji (Image J)]. If the desired image required stitching because the resultant image exceeded a tiling area of 1X by 1Y frame), IMARIS stitcher was used to properly align all images prior to conducting volumetric measures using the IMARIS software. Once the file was in ims format, it was dragged into the IMARIS software arena for 3D rendering and volume quantification. To quantify the ischemic lesion volume, we used 3D surface rendering to delineate the boundaries of the infarct based on its characteristic autofluorescence. In this context, a “surface” refers to a 3D region generated from voxels that share a defined intensity range. To avoid inadvertently capturing surrounding vasculature, we set the surface detail size to exceed the width of individual vessels, thereby excluding fine vascular structures. The ischemic volume was segmented using a rule-based thresholding workflow designed to minimize operator variability. For each imaging cohort, we generated an intensity histogram from a contralateral cortical region and applied an *a priori*-selected percentile as the global threshold for all samples. We confirmed the entire lesion was delineated by transforming the rendered surface into a semi-transparent mode, which enabled us to visually inspect and minutely adjust the intensity threshold if needed. Specifically, when heterogeneous fluorescence or nearby vessels produced multiple candidate surfaces, these were manually separated, and only the surface of corresponding to the infarct core was retained. Final ischemic volumes were obtained from the statistics and detailed analysis tabs to extract the volume of the selected region.

### 2.8 Tissue Sectioning and Cresyl Violet Staining

The brains (n=6) were rehydrated in increasing serial dilutions of methanol/water washes: 100%, 80%, and 60% (2 h at each stage, shaking, at room temperature) and transferred to 60% methanol for an overnight wash at room temperature. The following day, the brains were washed in methanol/water dilutions of 40% and 20% (2 h at each stage, shaking, at room temperature, RT) and transferred to 0.1 M PBS (overnight, at room temperature). The brains were then flash frozen in isopentane −30°C and coronally sectioned (50  μm) on a Leica cryostat and stained with cresyl violet. The sections were imaged on a FV300 Olympus confocal microscope (Evident Canada, Québec City, Canada), and the ischemic regions were outlined using ImageJ (National Institute of Health Research). Volumes were determined by multiplying the infarcted area for all coronal sections by the slice thickness.

### 2.9 Statistical Analysis

Statistical analyses were conducted using GraphPad Prism 10.5 for Mac (GraphPad software). Correlations were assessed through simple linear regression, and the coefficient of determination ($R^{2}$) was used to evaluate the proportion of variance across volumetric measures. One brain was excluded from analysis due to damage incurred during tissue sectioning. The brains were imaged before and after clearing on a grid (smallest squares were 1×1  mm, bold squares 10×10  mm), and percent changes in length, width, and height were measured. p-values are reported, with a p<0.05 considered statistically significant.

## 3 Results

### 3.1 Light Sheet Fluorescence Microscopy Enables Robust and Reproducible Infarct Volume Quantification

To enable 3D quantification of stroke lesions, whole brains were optically cleared and imaged using LSFM. Visual inspection revealed increased transparency and a noticeable reduction in brain size following clearing [Fig. 1(a)]. Morphometric analysis revealed moderate reductions in brain dimensions, with height, width, and length each decreasing by ∼10% to 30% [Fig. 1(b)]. These findings confirm that the clearing process induces measurable but consistent shrinkage, supporting the use of post-clearing volume estimates for comparative analyses. Following optical clearing, LSFM imaging revealed strong intrinsic autofluorescence within the ischemic lesion, enabling clear delineation of the infarct in three dimensions without the need for exogenous labeling [Figs. 1(c)–1(d), Video 1]. This allowed for comprehensive visualization of the ischemic area throughout the entire brain volume, capturing both lesion boundaries and spatial distribution. Notably, this approach additionally allowed for the visualization of strokes 4 weeks post-photothrombosis (Fig. S2 in the Supplementary Material). Furthermore, the ischemic volume can be detected across multiple imaging channels (Fig. S1 in the Supplementary Material); however, we chose to use the 561 laser with the 600/50-nm bandpass filter due to the strong signal-to-background ratio. To validate the accuracy of this technique and ensure that the inherent lower resolution in the z-plane does not compromise volumetric measurements, we compared infarct quantification of dorsal and sagittal imaging of the same cleared brains [Figs. 1(e)–1(g)]. Despite the differences in imaging orientation relative to the optical axis, infarct volumes derived from 2D projections in both planes were nearly identical (0.455 versus $0.453\;{\mathrm{mm}}^{3}$) [Figs. 1(e) and 1(g)]. Corresponding 3D reconstructions confirmed consistent lesion boundaries and morphology across views [Figs. 1(h) and 1(i)]. Linear regression analysis revealed a near-perfect correlation between sagittal and dorsal volume estimates ($r^{2}=0.999$, p<0.001) [Fig. 1(j)], demonstrating that LSFM provides reliable and orientation-independent volume measurements, unaffected by the z-plane resolution limitations.

### 3.2 LSFM-Derived Infarct Volumes Strongly Correlate with MRI and Histological Measurements

**Fig. 2:**
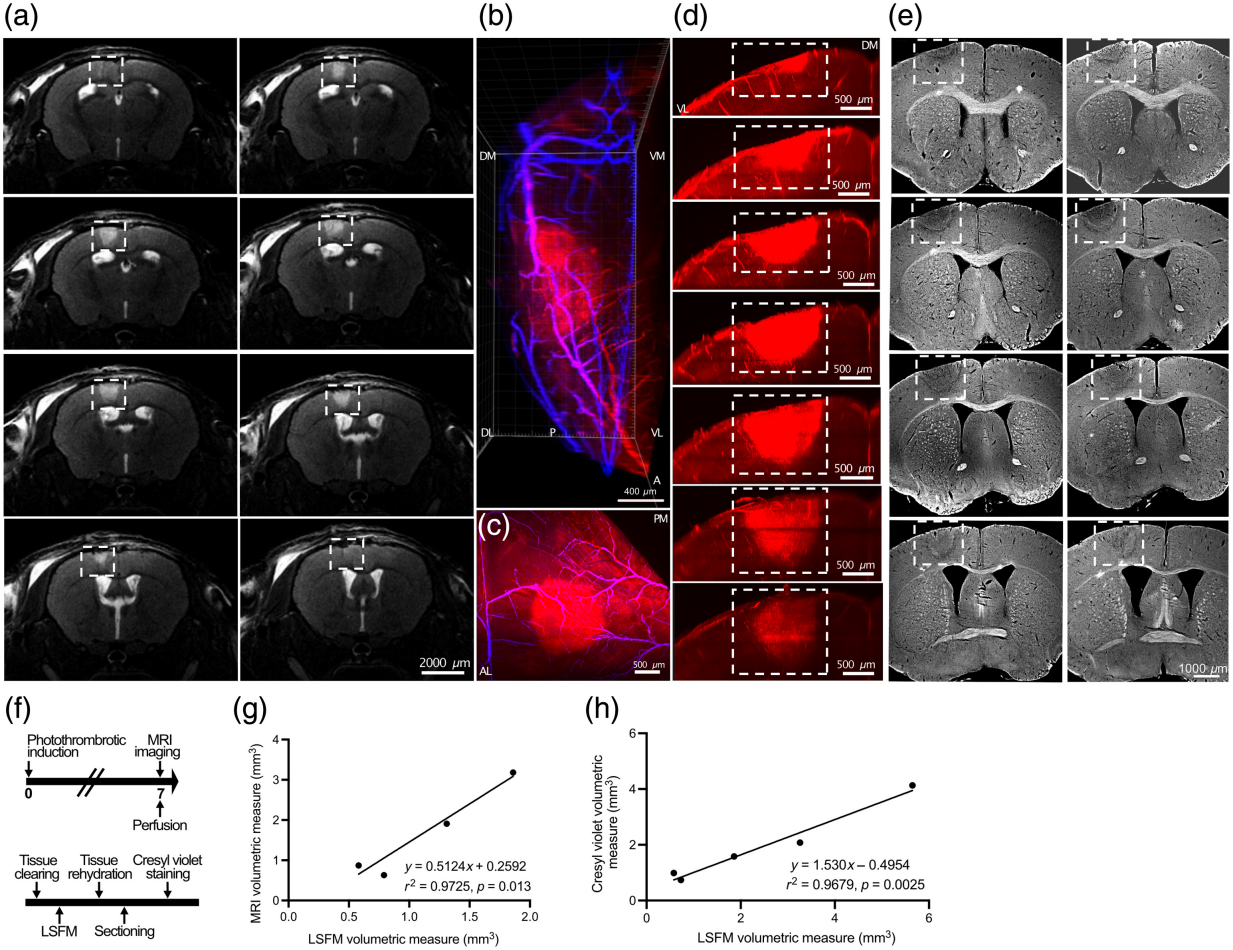
Cross-validation of ischemic volume quantification across MRI, LSFM, and cresyl violet histology. (a) T2-weighted MRI images of the coronal brain sections from a mouse at 7 days post-photothrombosis, showing a hyperintense signal corresponding to the ischemic lesion (dashed boxes). (b) 3D LSFM rendering of the autofluorescent stroke lesion and cerebrovasculature (Evans blue). (c) Merged LSFM image highlighting infarct autofluorescence (red) and vascular architecture (blue). (d) LSFM coronal sections taken at 200-μm intervals through the infarcted area, showing depth and spatial extent of the ischemic autofluorescence. (e) Corresponding coronal-cresyl-violet-stained sections taken at 200-μm intervals from the same brain shown in panels (a)–(d), identifying the lesion location and size. (f) Experimental workflow illustrating the timeline for MRI, LSFM, and histological processing. (g) and (h) Correlation analyses among volumetric measures obtained from different modalities: (g) LSFM versus MRI ($r^{2}=0.9725$, p=0.013) and (h) cresyl violet versus LSFM ($r^{2}=0.9679$, p=0.0025). N=1 brain was damaged during sectioning, and N=2 brains were added to compare cresyl violet and LSFM volumes. Scale bars: (a) 2000  μm, (b) 400  μm, (c) 500  μm, (d) 500  μm, and (e) 1000  μm. Orientations: D, dorsal; V, ventral; A, anterior; P, posterior; L, lateral; M, medial.

We next assessed how LSFM measurements compare to conventional imaging and histological methods. T2-weighted *in vivo* MRI and cresyl-violet-stained sections were acquired from the same brains used for LSFM (Fig. 2). LSFM clearly delineated ischemic lesions through endogenous autofluorescence, whereas vascular architecture was visualized via Evans blue labeling [Figs. 2(b) and 2(c)]. Serial LSFM sections at 200-μm intervals [Fig. 2(d)] closely matched the distribution of infarcts observed in T2-weighted *in vivo* MRI images [Fig. 2(a)] and cresyl-violet-stained sections [Fig. 2(e)]. Stroke sizes were particularly well-matched between LSFM and cresyl violet histology, reflecting the consistency of infarct localization and extent across these *ex vivo* methods. Volumetric measurements derived from all three modalities were strongly correlated: LSFM versus MRI ($r^{2}=0.9725$, p=0.013) [Fig. 2(g)] and cresyl violet versus LSFM ($r^{2}=0.9679$, p=0.0025) [Fig. 2(h)]. Although LSFM-derived infarct volumes tended to be smaller, due to tissue shrinkage associated with clearing, and MRI volumes slightly overestimated due to edema, the relative infarct volumes across mice remained tightly correlated across modalities. These results confirm that LSFM provides comparable measures of infarct volume to gold-standard techniques, with the added advantage of 3D visualization of the entire brain at cellular resolution.

### 3.3 LSFM Volumes Align with Early Impairment in Blood Flow Detected by Laser Speckle Imaging

**Fig. 3:**
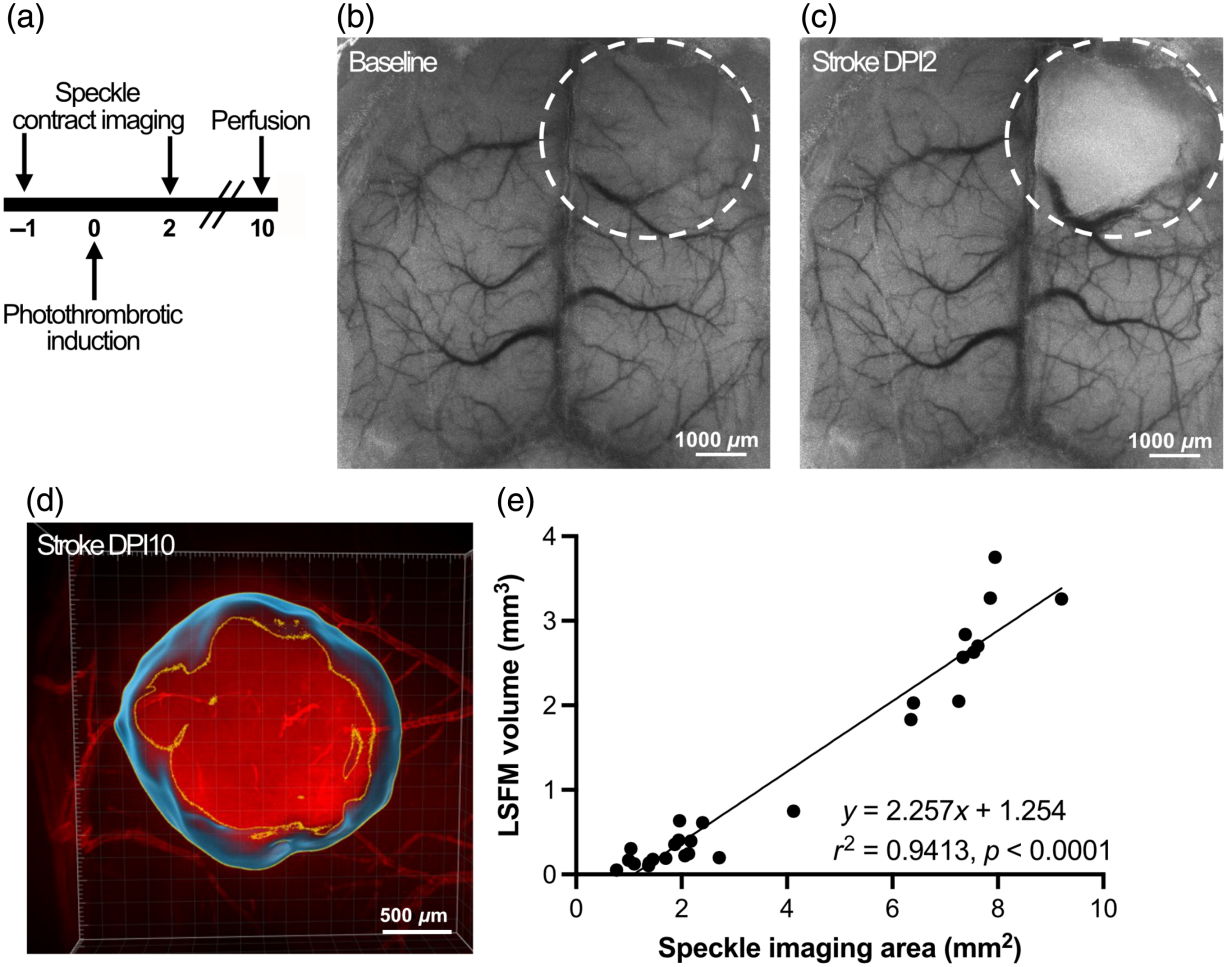
Correlation between LSCI- and LSFM-derived infarct volume following photothrombotic stroke. (a) Experimental timeline showing the sequence of baseline LSCI, photothrombotic stroke induction (day 0), post-stroke LSCI (day 2), and perfusion (day 10). (b) Baseline LSCI showing intact cortical vasculature. (c) LSCI performed 2 days post-stroke (DPI2) revealing a localized reduction in blood flow corresponding to the ischemic lesion (white dashed circle). (d) LSFM 3D reconstruction of the autofluorescent ischemic lesion day 10 post-injury (DPI10), showing volumetric delineation of infarcted tissue. (e) Linear regression demonstrating a strong correlation between infarct area measured by LSCI at DPI2 and infarct volume quantified by LSFM at DPI10 ($r^{2}=0.9413$, p<0.0001), indicating consistency across imaging modalities. Scale bars: (b) and (c) 1000  μm and (d) 500  μm.

To determine whether early post-stroke blood flow deficits align with eventual infarct volume, we performed LSCI before and 2 day after stroke induction of small and large induced photothrombotic strokes and compared the hypoperfused area to LSFM-derived lesion volumes 10 days post-injury (Fig. 3). The speckle-derived infarct area observed at day 2 was strongly correlated to the volume of autofluorescent infarcts at day 10 [$r^{2}=0.9413$, p<0.0001, Fig. 3(e)], demonstrating that early perfusion deficits predict eventual infarct size and further validate LSFM for post hoc volume quantification.

### 3.4 3D Rendering of the Stroke Lesion and Cerebrovasculature Using LSFM

**Fig. 4:**
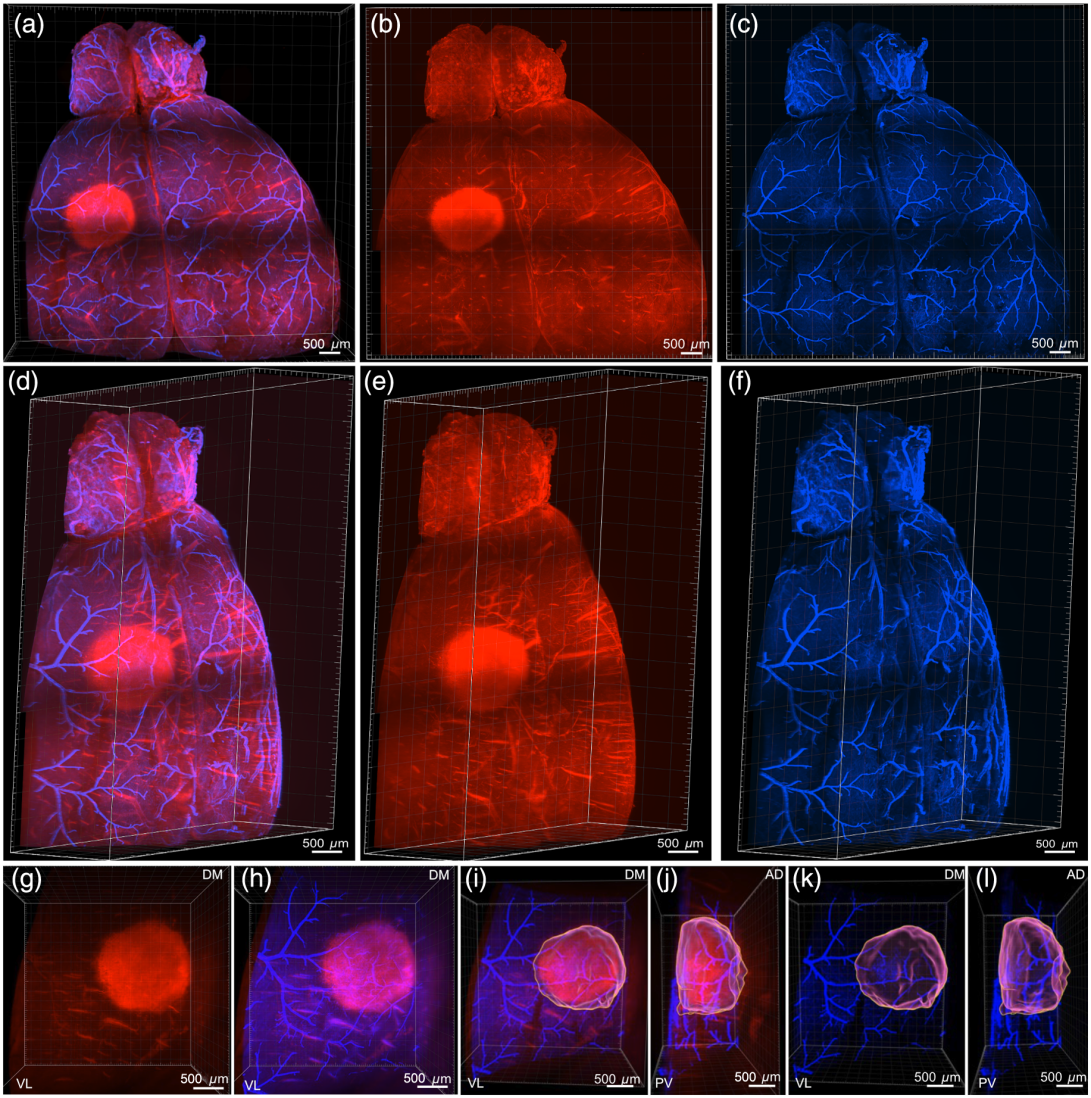
Volumetric light sheet imaging of a photothrombotic stroke and cerebrovascular labeling in the cleared mouse brain. (a) and (d) Whole-brain 3D reconstruction of a clarified mouse brain imaged via LSFM, displaying ischemic autofluorescence (red; imaged using a 561-nm fibered laser combined with a 600/50-nm band-pass emission filter) and the cerebrovasculature labeled with Evans blue (blue; using a 638-nm fibered laser combined with a 670/50-nm band-pass emission filter). (b) and (e) Whole-brain 3D reconstruction displaying only the ischemic autofluorescence. (c) and (f) Whole-brain 3D reconstructions displaying cerebrovasculature. (g) Magnified 3D view of a photothrombotic lesion showing stroke-specific autofluorescence. (h) Overlay of autofluorescence (red) and vascular signal (blue) within the stroke region, highlighting vascular disruption. (i) and (j) Orthogonal views (coronal and sagittal, respectively) of the ischemic lesion with volume rendering of the autofluorescent stroke core and surrounding vasculature. (k) and (l) Isolated visualization of the segmented ischemic volume from panels (i) and (j) emphasizing the lesion morphology relative to the vasculature. Scale bars: 500  μm. Orientations: D, dorsal; V, ventral; A, anterior; P, posterior; L, lateral; M, medial.

We leveraged LSFM to visualize the spatial relationship between the infarct core and cerebrovascular network at whole-brain resolution [Figs. 4(a) and 4(d)]. This approach was chosen because traditional histological methods often lack the ability to preserve and analyze intact vascular networks in three dimensions of the entire ischemic region, limiting our understanding of how microvascular injury propagates within the ischemic territory. Ischemic tissue exhibited strong autofluorescence (red) [Figs. 4(b) and 4(e)], whereas EB delineated the vasculature (blue) [Figs. 4(c) and 4(f)]. Orthogonal and rotated views of the infarct revealed a loss of EB signal in the microvasculature within the stroke core, whereas larger vessels remained visible [Figs. 4(h)–4(j)]. Volume renderings enabled precise segmentation of the lesion and highlighted its spatial relationship with penetrating vessels [Figs. 4(k) and 4(l)]. These 3D reconstructions demonstrate the ability of LSFM to integrate vascular data in a single preparation.

### 3.5 LSFM Detects Infarcts Reliably Across Fixation Protocols

**Fig. 5:**
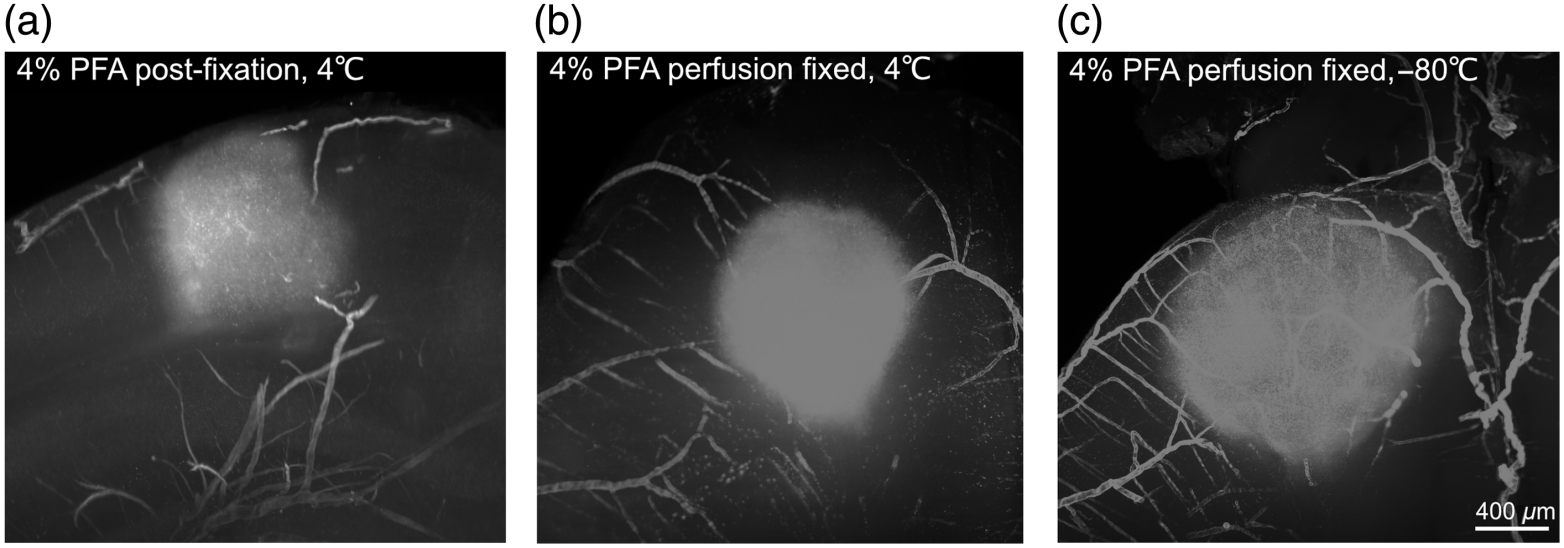
Autofluorescent visualization of photothrombotic lesions under different tissue preparation protocols. Representative LSFM images of clarified mouse brains displaying ischemic autofluorescence following photothrombosis imaged using a 561-nm fibered laser combined with a 600/50-nm band-pass emission filter were prepared under three fixation conditions: (a) 4% PFA post-fixation at 4°C without perfusion, (b) 4% PFA perfusion-fixed and stored at 4°C, and (c) 4% PFA perfusion-fixed, cryoprotected, and stored at −80°C. All methods enabled the detection of the ischemic core by intrinsic tissue autofluorescence. The lack of differences in lesion contrast and vascular preservation across conditions highlights the robustness of LSFM for ischemic lesion detection, regardless of fixation and storage methods. Scale bar: 400  μm.

Finally, we evaluated whether different tissue preparation methods affect infarct detection by LSFM (Fig. 5). The brains were processed under three common conditions: (i) post-fixed in 4% PFA at 4°C, (ii) perfusion-fixed and stored at 4°C, and (iii) perfusion-fixed, cryoprotected, and stored at −80°C (Fig. 5). Regardless of protocol, infarcts were clearly detected via autofluorescence, with no apparent differences in contrast or vascular preservation. This robustness across preparation methods further supports the utility of LSFM for consistent infarct visualization in archival tissue.

## 4 Discussion and Conclusion

LSFM offers an advanced optical platform for three-dimensional visualization of brain structure and pathology with high spatial resolution and minimal tissue distortion. In the context of stroke research, LSFM enables comprehensive imaging of ischemic lesions in optically cleared whole brains, providing an intact ischemic volume that is often lost in conventional two-dimensional histology. Here, we validated the utility of LSFM for quantifying photothrombotic infarcts by comparing volumetric measurements across imaging modalities, anatomical perspectives, and tissue preparation protocols.

We demonstrated that LSFM-derived infarct volumes are highly reproducible across imaging orientations, with dorsal and sagittal projections yielding nearly identical measurements. This was supported by strong spatial overlap in 3D reconstructions and a near-perfect correlation among views ($r^{2}=0.999$, p<0.001), underscoring the robustness of LSFM for reliable volumetric analysis in longitudinal and comparative studies, regardless of imaging angle (Fig. 1).

To benchmark LSFM against gold-standard methods, we cross-validated volumetric data with T2-weighted MRI and cresyl violet histology. All three techniques yielded strongly correlated infarct sizes, with $r^{2}$ values ranging from 0.968 to 0.972 (Fig. 2). In addition, we found that infarct areas identified by LSCI at 2 days post-injury strongly predicted LSFM-derived volumes obtained at 10 days post-stroke ($r^{2}=0.941$) (Fig. 3). Although these techniques provide valuable information, it is important to note that early MRI primarily reflects a combination of ischemic infarct as well as vasogenic and cytotoxic edema,^8^ whereas LSCI offers an indirect assessment of changes in cerebral blood flow.^9^ Furthermore, unlike MRI that can be used longitudinally for *in vivo* experiments, LSFM provides a volumetric measure at the experimental endpoint. Lesion volume in histology is typically calculated as the sum of infarct areas across sections, multiplied by the distance among them. Although histology remains the gold standard for lesion size quantification and is capable of providing spatial information about lesion location, it lacks 3D intact context, whereas LSFM adds value by providing volumetric continuity. Specifically, both histological and volume light sheet imaging require additional steps of registering regions of interest to brain atlases. However, such registration is more feasible for tissue volumes as the relative location of individual structures is fixed. One limitation of the technique is the tissue shrinkage inherent to the organic solvent-based clearing process, which may affect absolute volumetric accuracy. However, because the relative size of the lesion remains proportional to the overall brain volume, reliable comparisons can still be made across experimental groups. Our measurements revealed consistent shrinkage within each brain, with an approximate twofold reduction in total brain volume, further supporting the validity of comparative analyses. The strong intermodality and interorientation correlations observed in our study further support the robustness of LSFM-derived measurements, highlighting the accuracy and translational potential of LSFM in capturing stroke-related structural damage within controlled experimental contexts. Moreover, a practical consideration is file size with LSFM volumetric datasets being ∼2 to 3 GB per brain, compared with ∼25  MB per brain for MRI, a difference reflecting the higher spatial resolution of the LSFM acquisitions and imaging every 10  μm for LSFM rather than 200-μm intervals.

Variability in infarct size is photothrombotic stroke models can be caused by small fluctuations in beam diameter, illumination duration, and the proximity of the targeted region to cortical vessels, all contribute to the differences in the final ischemic volume.^13^ As this variability persists even under standardized induction parameters, volumetric quantification is critical for ensuring reliable comparison of ischemic severity across animals. In the present study, we intentionally incorporated a broad range of infarct sizes (both small and large lesions) to reflect this biological and technical reality. Importantly, this heterogeneity demonstrated that LSFM-based autofluorescent quantification remains robust across diverse infarct magnitudes, indicating that this method is well adapted for detecting meaningful differences both within and among experimental groups. Although variability was observed when comparing acute LSCI area measurements to LSFM volumetry, this likely reflects inherent differences between the type of signal acquired (surface perfusion deficit) and the 3D volume of structural tissue injury captured post-mortem.

LSFM offers a distinct advantage in preserving whole-brain architecture during tissue clearing and imaging, enabling high-resolution 3D visualization of infarct morphology across the entire brain. Unlike traditional section-based histology, LSFM maintains tissue integrity, allowing for accurate assessment of infarct depth, shape, and spatial relationships to the surrounding vasculature (Fig. 4). The ability to visualize and reconstruct the vasculature supports spatial registration with functional imaging data obtained through mesoscale wide-field imaging. Importantly, LSFM has become a mature and widely adopted technology, with robust protocols and commercially available platforms that enable reproducible, high-throughput whole-brain analysis in preclinical models.^16^

We assessed the flexibility of LSFM across tissue preparation workflows. The brains processed using post-fixation, perfusion, or cryoprotection and long-term storage all retained sufficient autofluorescence for infarct visualization (Fig. 5). The lack of contrast variability across conditions confirms LSFM’s adaptability to both fresh and archived samples, making it a practical tool for diverse experimental timelines.

The autofluorescence signal, detected across imaging channels (Fig. S1 in the Supplementary Material), used to delineate infarcts likely arises from a combination of metabolic and inflammatory processes, including the accumulation of FAD, lipofuscins, activated immune cells, and extracellular matrix (ECM) remodeling.^14^^,^^15^^,^^17^^–^^20^ Following a photothrombotic stroke, various cellular and biochemical changes can lead to increased autofluorescence detectable by LSFM. Key contributors include the accumulation of endogenous fluorophores such as ${\mathrm{FADH}}_{2}$ and lipofuscins,^14^ which accumulate in response to cellular stress or damage. The inflammatory response further contributes, with immune cells such as macrophages infiltrating the area and producing autofluorescent substances.^15^ Following inflammation, glial cell activation may lead to alterations in the metabolic activity of astrocytes of microglia or may result in an accumulation of autofluorescent materials, thereby contributing to the observed autofluorescence.^17^^–^^19^ Tissue damage and cellular breakdown also release autofluorescent components, such as lipofuscins, enhancing the signal in affected regions.^17^ Changes in blood–brain barrier permeability and alterations in the extracellular matrix may also contribute to the complex autofluorescence patterns observed post-stroke, offering insights into the underlying pathological processes. Finally, alterations in the ECM after brain injury, including the accumulation of advanced glycation end products, may further contribute to the increase in autofluorescent signals.^20^ Although autofluorescent signals were detectable across many spectral channels (Fig. S1 in the Supplementary Material), the 561-nm excitation/emission range consistently provided the clearest and most robust contrast between infarcted and healthy tissue. The exact contributors to the LSFM-detected signal remain complex; however, the signals offer a valuable, label-free biomarker for ischemic pathology, enabling visualization of infarcted regions without the need for exogenous staining. Furthermore, although autofluorescence within the infarct core is a time-dependent phenomenon, our results indicate that this signal remains robust within the first 10 days post-stroke, a window during which volumetric estimates are expected to be most accurate. Notably, we also observed clear autofluorescence in a photothrombotic lesion 4 weeks after stroke (Fig. S2 in the Supplementary Material), demonstrating that the signal can persist well beyond the acute phase and enabling the use of this method for later-stage or longitudinal assessments.

In summary, LSFM is a powerful tool for stroke research, offering precise, high-resolution imaging of intact ischemic lesions. Its compatibility with co-staining protocols, strong agreement with conventional modalities, and robustness across preparation methods position LSFM as a valuable platform for preclinical investigation of stroke mechanisms and therapies. As LSFM technology becomes increasingly accessible, its integration into stroke research workflows promises to enhance our understanding of injury dynamics and recovery pathways.

## Supplementary Material

10.1117/1.NPh.13.1.015009.s01

10.1117/1.NPh.13.1.015009.s1

## Data Availability

The data generated and analyzed for this article are publicly available in the Federated Research Data Repository at DOI: 10.20383/103.01147.
