# Supplementary material for: Quantifying photothrombotic ischemic volume in the intact brain using light sheet microscopy
Source: Neurophotonics. 2026 Feb 3;13(1):015009. doi: 10.1117/1.NPh.13.1.015009 (PMC12867488; doi:10.1117/1.NPh.13.1.015009)
Supplement: Supplementary file 1 [file NPh_013_015009_SD001.pdf]

Supplemental Figures and Legends:

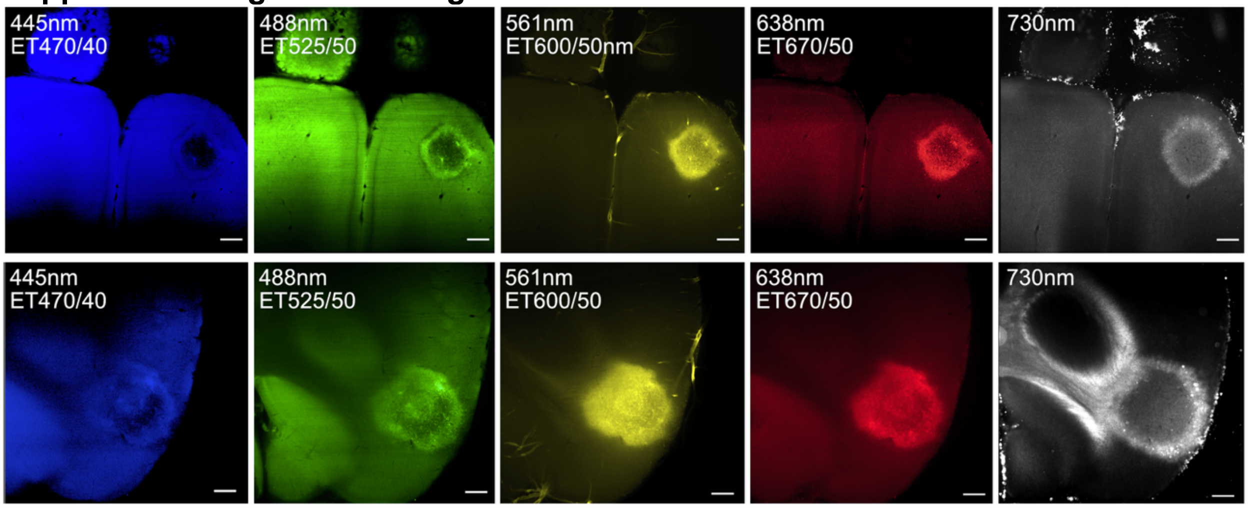

Supplemental Figure 1. Autofluorescent visualization of photothrombotic lesion across imaging channels. Representative light sheet fluorescence microscopy (LSFM) images of two ischemic regions following photothrombosis imaged using 445, 488, 561, 638, 730nm fibered lasers combined with their respective band-pass emission filters of brains post-fixed with PFA at 4 °C. Top: Prefrontal cortex; Bottom: Visual cortex. Scale bars: 300 µm.

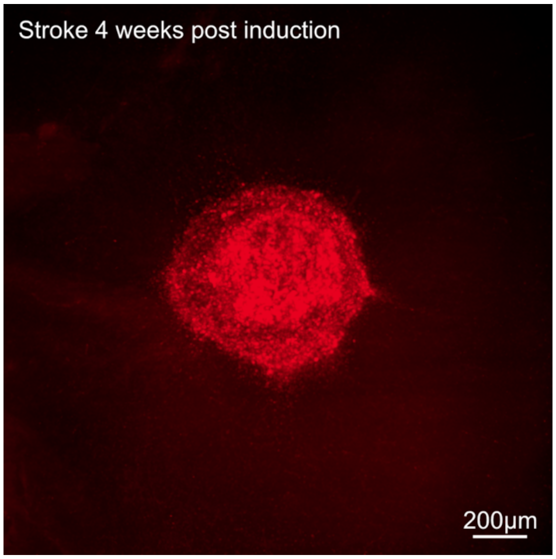

Supplemental Figure 2. Autofluorescent visualization of photothrombotic lesion at 4 weeks post injury. Representative light sheet fluorescence microscopy (LSFM) image of a clarified mouse brain displaying ischemic autofluorescence following photothrombosis imaged using a 561 nm fibered laser combined with a 600/50 nm band-pass emission filter post-fixed with PFA at 4 °C. Scale bar: 200 µm.

Supplemental video legend:

Supplemental Video 1: Three-dimensional LSFM rendering of a photothrombrotic infarct 7 days post-injury. This video shows a volumetric reconstruction of an optically cleared mouse brain 7 days after photothrombrotic stroke using LSFM. Intrinsic autofluorescence delineates the infarcted region, enabling clear visualization of lesion boundaries and reveals the spatial extent and cortical localization of the infarcted region, demonstrating the utility of LSFM for characterizing stroke pathology.
